# Identification of the Vessels Causing Myocardial Ischemia by a Synthesized 18-Lead Electrocardiogram Obtained After the Master Two-Step Exercise Test in a Patient With Effort Angina

**DOI:** 10.7759/cureus.47840

**Published:** 2023-10-27

**Authors:** Koji Takahashi, Daijiro Enomoto, Hiroe Morioka, Shigeki Uemura, Takafumi Okura

**Affiliations:** 1 Department of Cardiology, Yawatahama City General Hospital, Ehime, JPN

**Keywords:** u wave, synthesized 18-lead electrocardiography, st segment, master two-step exercise test, effort angina pectoris

## Abstract

A synthesized 18-lead electrocardiograph is a specialized technology that mathematically computes the virtual electrocardiographic waveforms of the right chest leads (V3R, V4R, and V5R) and posterior leads (V7, V8, and V9) based on a standard 12-lead electrocardiograph input without additional lead placement or techniques. Synthesized 18-lead electrocardiography is a useful test for the identification of the culprit coronary arteries in patients with ST-segment elevation myocardial infarction of the right ventricular wall or the posterior/lateral left ventricular wall, which are often missed on standard 12-lead electrocardiography. However, few studies have examined the usefulness of this modality during exercise stress testing. We present a case of a 78-year-old man with a two-month history of typical angina. The synthesized 18-lead electrocardiogram obtained just after the Master two-step exercise test revealed ST-segment shifts in multiple leads, including synthesized V4R, V5R, and V7-9 leads, and U-wave changes in some leads, including the synthesized V9 lead. The diagnosis of the culprit coronary arteries causing exercise-induced myocardial ischemia is discussed with reference to coronary angiographic findings. This modality could potentially increase the sensitivity and specificity for the detection of coronary artery disease and accurately pinpoint the site of the lesion. If an electrocardiograph can display a synthesized 18-lead electrocardiogram, it should be used when evaluating the waveform due to myocardial ischemia.

## Introduction

Myocardial ischemia in patients with effort angina is a dynamic condition that reflects a changing imbalance between blood supply and metabolic demand in the myocardium. Electrocardiography (ECG) under physical stress conditions, such as exercise, has long been in widespread clinical use [1]. However, a meta-analysis has clarified that exercise ECG without an imaging modality has poor sensitivity and specificity for detecting ischemic changes in stable coronary artery disease, and the latest guidelines limit the recommendations for using this test [2,3]. Nonetheless, exercise ECG has practical and economic advantages as compared to tests using imaging modalities, and any improvements in the technique of gathering and interpreting data will have a profound impact on clinical practice. For example, using the right chest leads in addition to the standard leads during exercise stress testing improves the sensitivity for detecting changes in ECG waveforms due to myocardial ischemia [4].

A synthesized 18-lead ECG is a specialized technology that mathematically computes the virtual waveforms of the right chest leads (V3R, V4R, and V5R) and posterior leads (V7, V8, and V9) based on a standard 12-lead ECG input without additional lead placement or techniques [5]. Several studies have reported the clinical usefulness of synthesized 18-lead ECG, including the identification of the culprit coronary artery in patients with ST-segment elevation myocardial infarction of the right ventricular wall (perfused by the right coronary artery (RCA)) or the posterior/lateral left ventricular wall (perfused by the left circumflex coronary artery (LCCA)), which are often missed on standard 12-lead ECG [6]. Additionally, a synthesized 18-lead ECG has been reported to be effective in identifying the origin of atrial and ventricular arrhythmias [7]. However, few studies have examined the usefulness of this ECG modality during exercise stress testing.

Herein, we report a case of a 78-year-old Japanese man with a 2-month history of typical angina. Synthesized 18-lead ECG with the Master two-step exercise test revealed ST-segment shifts in multiple leads, including synthesized V4R, V5R, and V7-9 leads and U-wave changes in some leads, leading to the identification of the culprit coronary arteries causing the exercise-induced myocardial ischemia.

The Master two-step exercise test, originally designed for diagnosing coronary heart disease, is performed at an exercise intensity of 6.8 metabolic equivalents for a 70-kg person. This test involves the use of a wooden two-step staircase measuring 22 inches in width, 30 inches in length, and 18 inches in height, with each step rising 9 inches. During the test, the patient rhythmically ascends and descends the two steps for a duration of 3 minutes [8].

## Case presentation

A 78-year-old man was admitted to our hospital because of effort-induced retrosternal chest heaviness for two months. The chest symptom had no associated symptoms, such as diaphoresis, and subsided within several minutes of taking a rest. He had hypertension, dyslipidemia, and current smoking as risk factors for cardiovascular disease and a history of transient ischemic attack. He was prescribed clopidogrel sulfate (25 mg twice daily), diltiazem hydrochloride (100 mg once daily), benidipine hydrochloride (8 mg once daily), olmesartan medoxomil (40 mg once daily), esaxerenone (2.5 mg once daily), and ezetimibe (10 mg once daily) at another clinic. He also had a history of statin intolerance.

**Figure 1:**
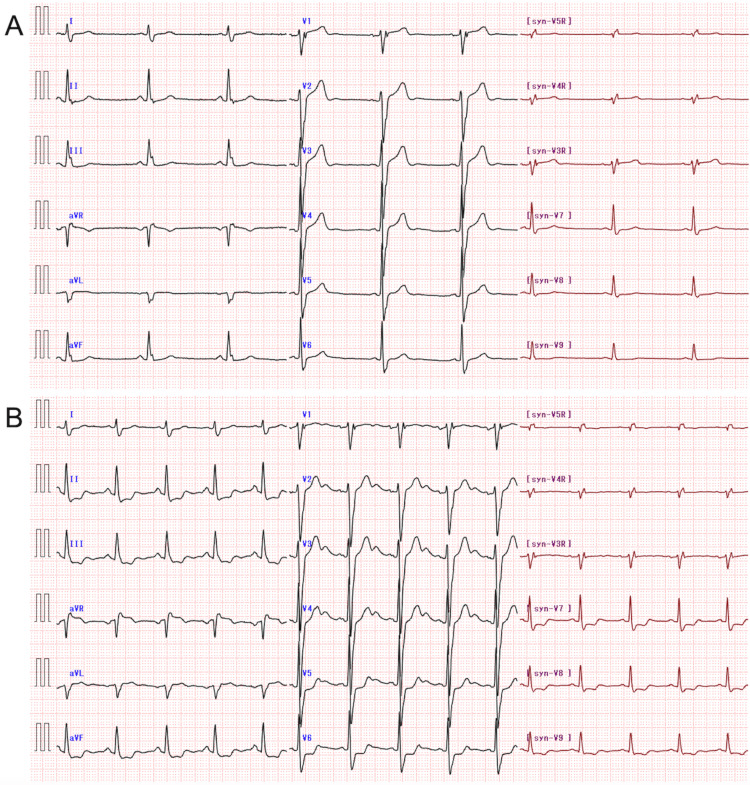
Synthesized 18-lead electrocardiography (ECG) (A) ECG performed immediately before the Master two-step exercise test shows a sinus rhythm with a heart rate of 51 beats/min and is within normal limits except for J waves in leads II, III, and aVF. (B) ECG performed 1 min after completing the Master two-step exercise test shows a heart rate of 86 beats/min, horizontal or downsloping ST-segment depression in leads II, III, aVF, V5–6, and synthesized V7–9, ST-segment elevation in leads aVR and aVL, prominent positive U waves in the precordial leads, and negative U waves in leads II, III, aVF, and synthesized V9. In addition, shallow ST-segment depression is shown in the synthesized V4R and V5R leads with low QRS voltage.

Vital signs were normal (temperature: 36.6 °C; pulse rate: 57 beats/min; blood pressure: 121/54 mmHg; oxygen saturation on room air: 98%). Physical examination revealed no abnormal findings, including the absence of significant heart murmurs. Blood tests revealed elevated high-sensitivity cardiac troponin I levels but normal brain natriuretic peptide levels (Table 1). In addition, blood tests for the risk of coronary artery disease revealed normal glucose and lipid profiles. Chest radiography revealed no pulmonary congestion. The synthesized 18-lead ECG performed at rest was within normal limits, except for J waves in leads II, III, and aVF (Figure 1A). Standard echocardiography revealed normal left ventricular wall motion, with an ejection fraction of 65%.

**Table 1:** Laboratory test values of the patient upon admission

Blood test parameter	Result	Reference value
C-reactive protein (mg/dL)	0.08	≤0.7
Creatine kinase (U/L)	68	≤190
Creatine kinase-MB (ng/mL)	1.1	≤5.0
hs-cTnI (pg/mL)	52.6	≤18.4
BNP (pg/mL)	11.2	≤18.4
LDL cholesterol (mg/dL)	110	≤140
HDL cholesterol (mg/dL)	43	35–70
Triglyceride (mg/dL)	128	Fasting: 45–150
Glucose (mg/dL)	90	Fasting: 70–109
Glycated hemoglobin A1c (%)	5.6	4.6–6.2
eGFR (mL/min per 1.73 m^2^)	62	90–120
White blood cells (/μL)	7,700	4,000–8,500
Red blood cells (/μL)	388 × 10^4^	400 × 10^4^–550 × 10^4^
Hemoglobin (g/dL)	12.5	13.0–17.5
Platelet (/μL)	23.9 × 10^4^	14 × 10^4^–40 × 10^4^

**Figure 2:**
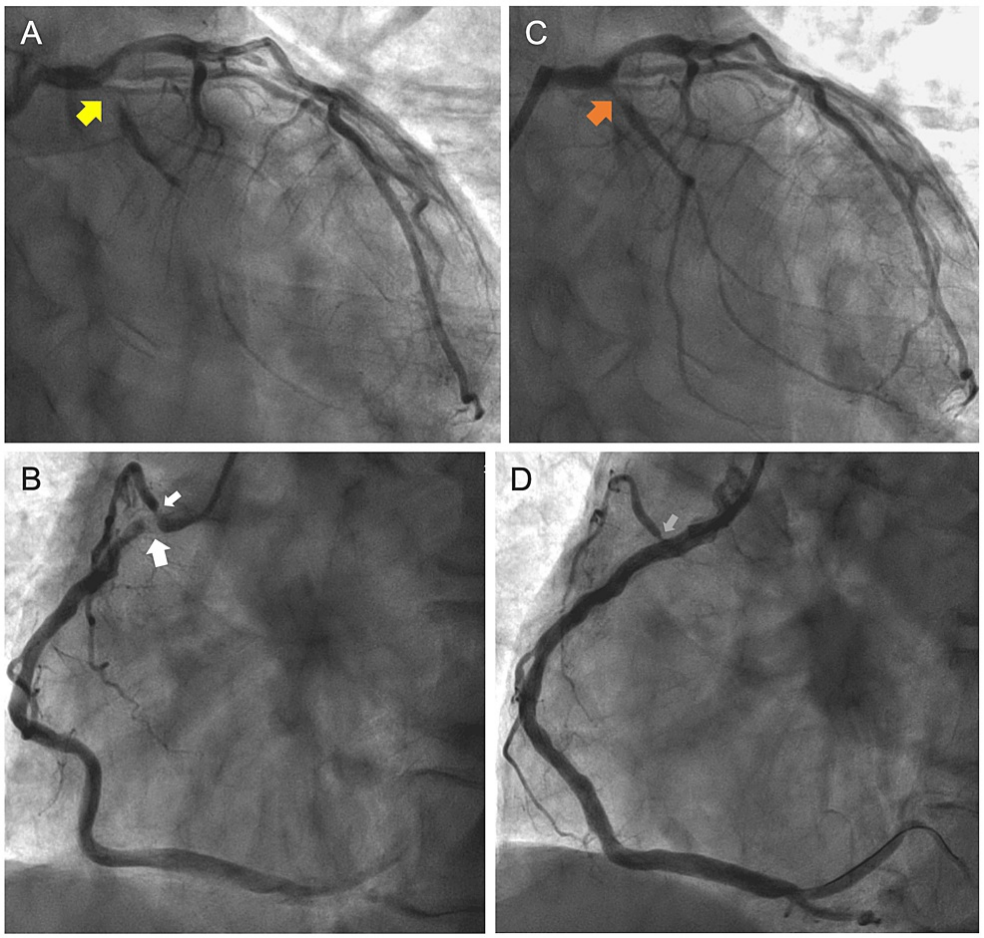
Coronary angiography Coronary angiograms reveal severe narrowing without thrombi in the proximal left circumflex coronary artery (LCCA) (yellow arrow) and proximal right coronary artery (RCA) (large white arrow), including the right ventricular branch (small white arrow); however, only moderate lesions with no significant stenosis are found in the left anterior descending coronary artery (A and B). An intravascular ultrasonography-guided 3.0 × 20 mm everolimus-eluting platinum–chromium coronary stent is successfully deployed to the proximal RCA and post-dilated using a 3.0-mm noncompliant balloon without residual stenosis (D), although the ostium of the right ventricular branch remains stenotic (gray arrow). In contrast, a 2.5 × 20-mm drug-coated balloon is dilated at the proximal LCCA after pre-dilation using a 2.5-mm noncompliant balloon, resulting in residual stenosis (orange arrow) (C). The images in panels A and B represent the anteroposterior and 30° caudal views, and those in panels C and D represent the 50° left anterior oblique view.

Due to the presence of stable angina and normal rest ECG, synthesized 18-lead ECG with the Master two-step exercise test was performed on the same day, despite observing slightly elevated high-sensitivity cardiac troponin I level. This exercise ECG revealed significant ST-segment shifts and U-wave changes in multiple leads, suggesting the two-vessel, the RCA and LCCA, disease, but not high-risk coronary artery disease such as left main coronary artery (LMCA)/LMCA-equivalent disease and three-vessel disease (Figure 1B). By shared decision-making between our healthcare providers and the patient and his family based on the symptoms and aforementioned test results, the patient and his family desired early coronary angiography as anatomic testing, but not coronary computed tomography or other functional testing such as stress echocardiography and nuclear testing. Coronary angiography was performed three days after the Master two-step exercise ECG, which revealed 99% stenosis at the proximal LCCA and proximal RCA (Figure 2). Subsequently, intravascular ultrasonography-guided coronary intervention was performed for both lesions.

## Discussion

The standard criteria for exercise ECG test positivity include horizontal or down-sloping ST depression ≥0.1 mV below the PQ baseline at 60-80 ms after the J point in two or more anatomically contiguous leads [9]. In addition, a positive ST-segment elevation is defined as an additional increase of ≥0.1 mV at 60 ms after the J point in two or more anatomically contiguous leads compared with the baseline tracing. Transient U-wave inversion is defined as the presence of a discrete negative deflection of ≥0.05 mV within the TP segment. Remarkable postexercise ECG findings in our patient were the horizontal or downsloping ST-segment depression in leads II, III, aVF, V5-6, and synthesized V7-9; ST-segment elevation in leads aVR and aVL; prominent positive U waves in the precordial leads; and negative U waves in leads II, III, aVF, and synthesized V9. In addition, a shallow ST-segment depression was observed in the synthesized V4R and V5R leads with a low QRS voltage. Thus, even a standard 12-lead ECG yielded positive test results and suggested the presence of transient ischemia in the RCA and LCCA territories, as confirmed by coronary angiography. However, the findings in the synthesized 18-lead ECG were more direct and revealing.

In patients with infarcts of the right ventricular wall, which is perfused by the RCA, or the posterior/lateral left ventricular wall, which is perfused by the LCCA, the diagnosis of ST-segment elevation myocardial infarction by synthesized 18-lead ECG is useful for identifying the site of infarction, which can often be missed on standard 12-lead ECG [5,6]. However, few studies have examined the usefulness of synthesized 18-lead ECG during exercise stress testing in patients with effort angina. In general, exercise-induced myocardial ischemia in the left ventricle leads to ST-segment depression on standard 12-lead ECG, and no correlation exists between the diseased coronary artery and the leads in which ST-segment depression occurs [10]. That is, exercise-induced ST-segment depression due to myocardial ischemia is likely to occur in leads II, III, aVF, and V-6, regardless of the culprit vessel. Conversely, exercise-induced transient U-wave inversion helps locate the culprit vessel [11]. In our patient, a negative U-wave in lead III may have indicated ischemia in the inferior wall of the left ventricle perfused by the dominant RCA [12,13]. Similarly, negative U waves in the synthesized V9 lead and reciprocal prominent U waves in the precordial leads may indicate posterior/lateral wall ischemia perfused by the nondominant LCCA [14]. Moreover, ST-segment changes in synthesized V4R and V5R leads, but not in the synthesized V3R leads, may identify LCCA disease [4]. However, no ECG manifestations suggestive of ischemia in the territory of the left anterior descending coronary artery, such as a leftward QRS axis shift or negative U waves in the precordial leads [15-17], were found.

Guideline-directed management of patients with stable coronary artery disease is recommended worldwide [2,3,18]. First, the likelihood that a patient actually has obstructive coronary artery disease must be estimated based on symptoms, age, and sex. Our 78-year-old male patient with typical angina had a high suspected pretest probability of obstructive coronary artery disease (European Society of Cardiology-Diamond and Forrester pretest probability of 89%) [19]. Patients with an intermediate or high pretest probability should undergo noninvasive diagnostic tests, functional testing, and/or anatomical testing for further evaluation and management. In patients with a high pretest probability of coronary artery disease, such as our patient, stress imaging testing for risk assessment, including exercise ECG, stress echocardiography, or nuclear stress testing, is preferable as an initial imaging test. In this setting, if patients are still able to exercise and cooperate with testing, an exercise ECG is the lowest-cost procedure used in the diagnostic evaluation when compared with other stress imaging tests. However, the risks associated with exercise testing, such as the potential to induce unstable angina or worsen a patient’s hemodynamic status, should always be considered. Exercise ECG has a higher risk of false-negative and false-positive test results than diagnostic imaging tests and has limited power to rule in or rule out obstructive coronary artery disease. Furthermore, an exercise ECG is of no diagnostic value in patients with ECG abnormalities that prevent interpretation of the ST-segment changes during stress, such as left bundle branch block. Therefore, the latest guidelines recommend the use of stress imaging diagnostic tests, such as pharmacological stress echocardiography and nuclear testing, rather than exercise ECG, as the initial test to diagnose obstructive coronary artery disease. The performance of exercise ECG is adequate only when the pretest probability is extremely high or low [2,3]. An exercise ECG using a Master two-step exercise stress test was performed first in our patient who was capable of exercise and had no contraindications for performing the test, although symptom-limited graded exercise ECG using a treadmill and ergometer, rather than a Master two-step test, is recommended in the guidelines. The Master two-step exercise stress test is now used for clinical purposes other than the detection of myocardial ischemia [8].

Invasive coronary angiography is preferred if a noninvasive imaging test demonstrates findings suggestive of LMCA/LMCA-equivalent disease. Our patient, who had only two-vessel disease (RCA and LCCA), not LMCA/LMCA-equivalent disease, underwent this test after shared decision-making. Stress-induced ST segment elevation in lead aVR, as observed in this patient, is commonly considered a sign of LMCA occlusion [20]. However, lead aVR, of which the positive pole is oriented to the right upper side of the heart, usually provides a mirror image of leads oriented leftward. When considered anatomically contiguous manner, the ST-segment shift in leads aVL, I, and −aVR (lead aVR with reversed polarity) indicates left superior basal myocardial ischemia [12]. In this patient, the postexercise ECG revealed ST-segment elevation in lead aVL, an isoelectric ST segment in lead I, and ST-segment depression in lead −aVR. These discrepancies in ST-segment shifts in leads aVL, I, and −aVR may indicate that exercise did not induce myocardial ischemia in the left superior basal region. ST-segment elevation in any lead is usually associated with reciprocal ST-segment depression in leads with a positive pole directed opposite to the leads that show ST-segment elevation and vice versa. ST-segment elevation in a lead with its positive pole located to the right and superiorly, such as lead aVR, is the reciprocal of and is similar in meaning to ST-segment depression in a lead with its positive pole located to the left and inferiorly such as lead II. Similarly, ST-segment elevation in lead aVL is the reciprocal of and is similar in meaning to ST-segment depression in lead III.

## Conclusions

We herein reported on a patient with transient ischemia in lesions perfused by RCA and LCCA, in which synthesized 18-lead ECG obtained just after the Master two-step exercise test pointed out the correct culprit arteries. This modality could potentially increase the sensitivity and specificity for the detection of coronary artery disease and accurately pinpoint the site of the lesion although it will need to undergo validation in a larger population before clinical implementation. If an electrocardiograph can display a synthesized 18-lead ECG, it should be used when evaluating myocardial ischemia-induced ECG waveform. It is important to become familiar with the 18-lead ECG on a regular basis.
